# Differential Expression of MicroRNAs in Silent and Functioning Corticotroph Tumors

**DOI:** 10.3390/jcm9061838

**Published:** 2020-06-12

**Authors:** Araceli García-Martínez, Antonio C. Fuentes-Fayos, Carmen Fajardo, Cristina Lamas, Rosa Cámara, Beatriz López-Muñoz, Ignacio Aranda, Raúl M. Luque, Antonio Picó

**Affiliations:** 1 Research Laboratory, Alicante General University Hospital-Institute for Health and Biomedical Research (ISABIAL), CIBERER, 03010 Alicante, Spain; araceli86gm@gmail.com; 2 Maimonides Institute for Biomedical Research of Cordoba (IMIBIC), 14004 Córdoba, Spain; b22fufaa@uco.es (A.C.F.-F.); raul.luque@uco.es (R.M.L.); 3 Department of Cell Biology Physiology and Immunology, University of Cordoba, 14004 Córdoba, Spain; 4 Hospital Universitario Reina Sofía, 14004 Córdoba, Spain; 5 CIBER Physiopathology of Obesity and Nutrition (CIBERobn), 14004 Córdoba, Spain; 6 Endocrinology Department, Hospital Universitario de La Ribera, 46600 Alzira, Valencia, Spain; fajardo_carmon@gva.es; 7 Endocrinology Department, Complejo Hospitalario Universitario de Albacete, 02006 Albacete, Spain; clamaso@sescam.jccm.es; 8 Endocrinology Department, Hospital Universitario y Politécnico La Fe, 46026 Valencia, Spain; rosacamaragomez@gmail.com; 9 Endocrinology Department, Alicante General University Hospital-ISABIAL, 03010 Alicante, Spain; bealopezmz@gmail.com; 10 Pathology Department, Alicante General University Hospital-ISABIAL, 03010 Alicante, Spain; ignaranda@gmail.com; 11 Endocrinology Department, Alicante General University Hospital-ISABIAL, Miguel Hernández University, CIBERER, 03010 Alicante, Spain

**Keywords:** pituitary neuroendocrine tumors, corticotroph tumors, miRNA, transcriptional regulation

## Abstract

The potential role of miRNAs in the silencing mechanisms of pituitary neuroendocrine tumors (PitNETs) has not been addressed. The aim of the present study was to evaluate the expression levels and the potential associated role of some miRNAs, pathways, and transcription factors in the silencing mechanisms of corticotroph tumors (CTs). Accordingly, the expression of miR-375, miR-383, miR-488, miR-200a and miR-103; of *PKA*, *MAP3K8*, *MEK*, *MAPK3*, *NGFIB*, *NURR1*, *PITX1*, and *STAT3* were analyzed via qRT-PCR in 23 silent and 24 functioning CTs. miR-200a and miR-103 showed significantly higher expression in silent than in functioning CTs, even after eliminating the bias of tumor size, therefore enabling the differentiation between the two variants. Additionally, miR-383 correlated negatively with *TBX19* in silent CTs, a transcription factor related with the processing of *POMC* that can participate in the silencing mechanisms of CTs. Finally, the gene expression levels of miR-488, miR-200a, and miR-103 were significantly higher in macroadenomas (functioning and silent) than in microadenomas. The evidence from this study indicates that miRNAs could be involved in the pathophysiology of CTs. The translational implications of these findings suggest that pharmacological treatments specifically targeting these miRNAs could become a promising therapeutic option for these patients.

## 1. Introduction

Corticotroph tumors (CTs) are a pituitary neuroendocrine tumor (PitNET) subtype characterized by the autonomous hypersecretion of adrenocorticotropic hormone (ACTH), which gives rise to an associated endocrine clinical syndrome called Cushing disease [1]. CTs associated with this disease are called functioning CTs. On the other hand, there are other ACTH-secreting pituitary tumors named silent CTs, which are not associated with any of the most important clinical and biochemical features of Cushing disease (i.e., hypercortisolemia) [2]. Together, CTs represent up to 17% of all PitNETs [3,4] of which approximately 45% are silent CTs [5,6]. However, the silencing mechanisms and pathogenesis of CTs remain unclear. The most feasible hypothesis lies in post-transcriptional dysregulation of the *POMC* gene, a precursor of ACTH [7,8,9,10,11]. However, other genetic or epigenetic alterations such as DNA methylation or microRNA (miRNA) regulation may influence the clinical behavior of this PitNET subtype. Indeed, we have recently published the effect of the DNA methylation of some tumor suppressor genes in the functionality of the CTs [12], but to the best of our knowledge, no studies have been conducted to date in order to determine the presence and potential role of miRNAs in CTs.

As small non-coding RNA molecules, miRNAs [13] are involved in the post-transcriptional regulation of gene expression through their inhibitory action on messenger RNA. In the mouse corticotroph AtT-20 cell line, Zhang et al. [14] demonstrated that some miRNAs participate in the regulation of *POMC* expression, which is activated by the hypothalamic release of the corticotropin-releasing hormone (CRH) in response to environmental stimuli. CRH activates the *PKA-MAP3K8-MEK-ERK1/2-NGFIB* pathway together with *POMC* transcription, while miR-375 negatively regulates this pathway inhibiting the *MAP3K8* expression [14]. In addition, other miRNAs such as miR-488, miR-383, and miR-200a have been reported as capable of inhibiting hypothalamic *POMC* expression [15]. Finally, it has been described in mice a transcriptional regulation of the *POMC* expression through the activation of the *POMC* promoter by transcription factors such as the bicoid-type homeodomain transcription factor pituitary homeobox 1 (*PITX1*) and the signal transducer and activator of transcription 3 (*STAT3)*, which is activated by cytokines and presents two binding sites on the *POMC* promoter [16].

Although some authors have also been reporting changes (up- and down-regulation) in the expression of certain miRNAs in human pituitary tumors [17,18,19,20,21,22], few have addressed the presence and potential association of miRNAs in the silencing mechanisms of silent PitNETs, specifically the CT [23]. Therefore, the aim of the present study was to evaluate, for the first time, the expression levels and potential association of miR-375, miR-383, miR-488, miR-200a, and miR-103 and the *PKA-MAP3K8-MEK-ERK1/2-NGFIB* pathway plus the transcription factors *PITX1* and *STAT3* in the silencing mechanisms of CTs.

## 2. Materials and Methods 

### 2.1. Patients and Samples

We selected 47 tumors of the corticotroph line, of which 23 were silent CTs, from our collection of PitNETs according to the quantity and quality of the genetic material. We collected anonymized clinical, radiological, immunohistochemical, and molecular data from the Spanish Molecular Registry of PitNETs (REMAH) database, a multicenter clinical-basic collaborative project of the Spanish Society of Endocrinology [24]. Identification of the PitNET subtypes was performed in light of the clinical and dominant relative gene expression of the pituitary-specific hormone genes and the specific transcription factor, Tpit, as previously described [5,6,25]. We defined micro- and macroadenomas according to maximum tumor diameter (MTD) and invasiveness according to Knosp classification [26]. Patients’ demographic and clinical characteristics are shown in Table 1 and Supplementary Table S1.

The study was performed in compliance with the Declaration of Helsinki and other applicable legislation. The local ethics committee (CEIC General University Hospital of Alicante) approved the protocol. None of the patients were from a vulnerable population, and all donors or their relatives provided written informed consent.

### 2.2. DNA and RNA Extraction and cDNA Synthesis

All molecular studies took place in the laboratory of the research institute of the General University Hospital of Alicante (ISABIAL).

All samples were preserved immediately after surgery in RNAlater solution at 4 °C for 24 h and then stored at −20 °C. Biological samples were disintegrated in the TissueLyser (Qiagen, Hilden, Germany). The AllPrep DNA/RNA/Protein Kit (Qiagen) was employed for manual RNA and miRNA extraction and their concentration and purity were measured in the Nanodrop spectrophotometer (Thermo Scientific, Waltham, MA, USA).

For the RNA retro-transcription reaction, 2 μg of RNA were used in a total volume of 20 μL, employing the High-Capacity cDNA Reverse Transcription Kit (Applied Biosystems, California, CA, USA). For the miRNA retro-transcription reaction, 10 ng of miRNA were used in a total volume of 15 μL, by means of the TaqMan MicroRNA Reverse Transcription Kit (Applied Biosystems) with RT primers (5X) of each miRNA of interest. 

### 2.3. Quantitative Real-Time Polymerase Chain Reaction (qRT-PCR)

We performed qRT-PCR following the manufacturer’s instructions in the QuantStudio 12K (Life Technologies, California, CA, USA). The following genes and miRNAs were analyzed: *PKA*, *MAP3K8*, *MEK*, *MAPK3*, *NGFIB*, *NURR1*, *PITX1*, *STAT3*, miR-375, miR-383, miR-488, miR-200a, and miR-103.

For the quantification of miRNA expression, we used TaqMan Fast Advanced PCR Master Mix and assays based on hydrolysis probes (TaqMan Gene Expression Assays, Life Technologies). We selected the following assays (20X): has-miR-375 (000564), has-miR-200a (000502), has-miR-383 (000573), has-miR-103 (000439), and has-miR-488 (002357). As endogenous controls, we used has-miR-361 (000554) and has-miR-186 (002285). The qRT-PCR protocol was as follows: 95 °C for 20 s, 40 cycles of 95 °C for 1 s, and 60 °C for 20 s.

For the quantification of RNA expression, we used PrimeTime Gene Expression Master Mix and PrimeTime Std qPCR Assays (Integrated DNA Technologies, Iowa, USA.). The primer/probe concentration in the reaction was 1X concentrated, meaning the final concentration was 500 nM primers and 250 nM probes. The selected probes were *PRKACA (PKA)* (Hs.PT.58.2681637), *NR4A2 (NURR1)* (Hs.PT.58.704850), *STAT3* (Hs.PT.58.20367494), *MAP2K1 (MEK)* (Hs.PT.58.19983600), *MAP3K8* (Hs.PT.58.2108922), *MAPK3 (ERK1)* (Hs.PT.58.24504720), *PITX1* (Hs.PT.58.1678716), and *NR4A1 (NGFIB)* (Hs.PT.58.39997829). Endogenous controls were *PGK1* (Hs.PT.58v.606641) and *TBP* (Hs.PT.39a.22214825). The qRT-PCR protocol for IDT’s Fast Cycling was as follows: 95 °C for 3 min, 35 to 45 cycles of 95 °C for 5 s, and 60 °C for 30 s. For the quantification of *NEUROD1* and *TBX19* mRNA expression, which our group carried out in a previous study [11], we used TaqMan Fast Advanced PCR Master Mix and an assay based on hydrolysis probes: *NEUROD1* (Hs01922995_s1) and *TBX19* (Hs00193027_m1) (TaqMan Gene Expression Assay, Life Technologies, California, CA, USA) and the same fast cycling protocol described above.

A pool of RNA/miRNA from nine normal pituitary samples obtained from autopsies served as a calibrator. All samples were analyzed in duplicate. We included negative controls to check that the probes did not amplify genomic DNA contaminant. The relative differences in gene expression were expressed as fold change and were obtained with the 2^−ΔΔCt^ method (SDS software, Applied Biosystems).

### 2.4. Bioinformatics Analysis: miRNA Target Prediction Methods

We used three programs to predict the putative miRNA targets: miRBase (http://www.mirbase.org/), Targetscan (http://www.targetscan.org/vert_72/), and PicTar (https://pictar.mdc-berlin.de/cgi-bin/PicTar_vertebrate.cgi) (Supplementary Table S2). 

### 2.5. Statistical Analysis

We expressed qualitative variables, including PitNET subtypes, as absolute and relative frequencies. Participant age and MTD were expressed as mean ± standard deviation (SD). We used the Shapiro–Wilk test to assess the distribution of the molecular variables (fold change (FC)), the Kruskal–Wallis and Mann–Whitney tests to compare qualitative variables and non-parametric quantitative molecular variables, and the student’s *t* and ANOVA tests when the quantitative variables showed a normal distribution. We used Spearman and Pearson correlation tests to compare quantitative variables. The area under the receiver operating characteristics (ROC) curve (AUC) was used to assess the sensitivity and specificity of the miRNA biomarkers. *p* Values of less than 0.05 were considered statistically significant. Statistical analysis and ROC curves were performed with SPSS 24.0 software (IBM Software, Ohio, OH, USA). All other figures were performed using GraphPad Prism 6 (GraphPad Software Inc., California, CA, USA).

## 3. Results

### 3.1. miR200a and miR-103 as Potential Diagnostic Biomarkers of Functionality in CTs

**Figure 1:**
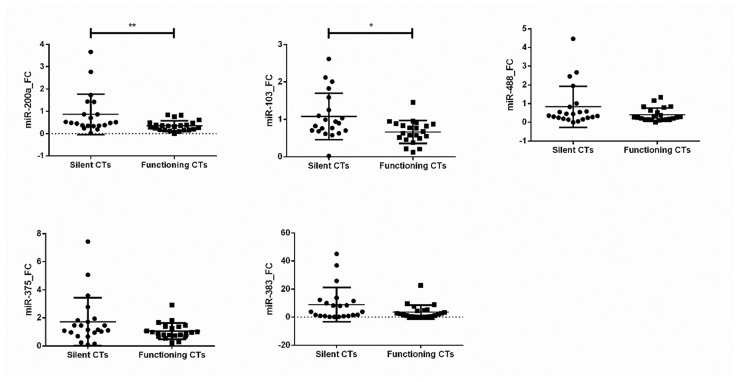
Gene expression differences (fold change) of miR-200a, miR-103, miR-488, miR-375 and miR-383 between silent CTs (corticotroph tumors) and functioning CTs. * *p* < 0.05; ** *p* < 0.01.

The miRNAs studied appeared up- or down-regulated in both functioning and silent CTs compared with normal pituitary glands, but only miR-200a and miR-103 showed significant differences between functioning CTs and their silent counterparts (Figure 1).

Eliminating the bias of tumor size, significant differences held between macro silent CTs and macro functioning CTs (*p* = 0.049 and *p* = 0.05, respectively). Therefore, we performed a ROC curve analysis in order to determine the potential usefulness of the expression of these two miRNAs in discriminating silent CTs from functioning CTs. Expression of both miR-200a and miR-103 enabled the differentiation between the two variants (AUC = 0.739, 95% CI 0.592 to 0.887, *p* = 0.007; AUC = 0.727, 95% CI 0.574 to 0.880, *p* = 0.011, respectively) (Supplementary Figure S1).

**Figure 2:**
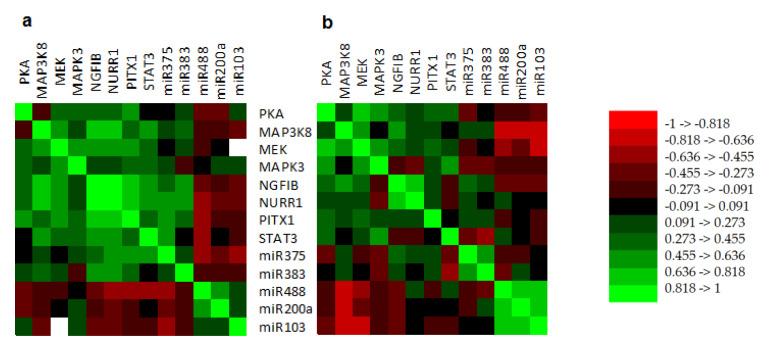
HeatMap between gene and pathways involved in the silencing of corticotroph tumors. (**a**) Functioning corticotroph tumors (*n* = 24); (**b**) Silent corticotroph tumors (*n* = 23). Legend of numbers associated with colors refers to the coefficients of correlation.

Additionally, in silent CTs, there were strong negative correlations between: (1) the expression of MAP3K8 and miR-488 (rho = −0.747, *p* < 0.001), miR-200a (rho = −0.686, *p* = 0.001), and miR-103 (rho = −0.782, *p* < 0.001); (2) the expression of MEK and miR-488 (rho = −0.605, *p* = 0.006) and miR-103 (rho = −0.665, *p* = 0.002), and (3) the expression of STAT3 and miR-383 (rho = −0.544, *p* = 0.016). In functioning CTs, only the miR-488 expression showed a negative correlation with the expression of NURR1 (rho = −0.531, *p* = 0.016), PITX1 (rho = −0.478, *p* = 0.033) and STAT3 (rho = −0.553, *p* = 0.011) (Figure 2).

### 3.2. TBX19 Could Be a Putative Target Gene of miR-383 

**Figure 3:**
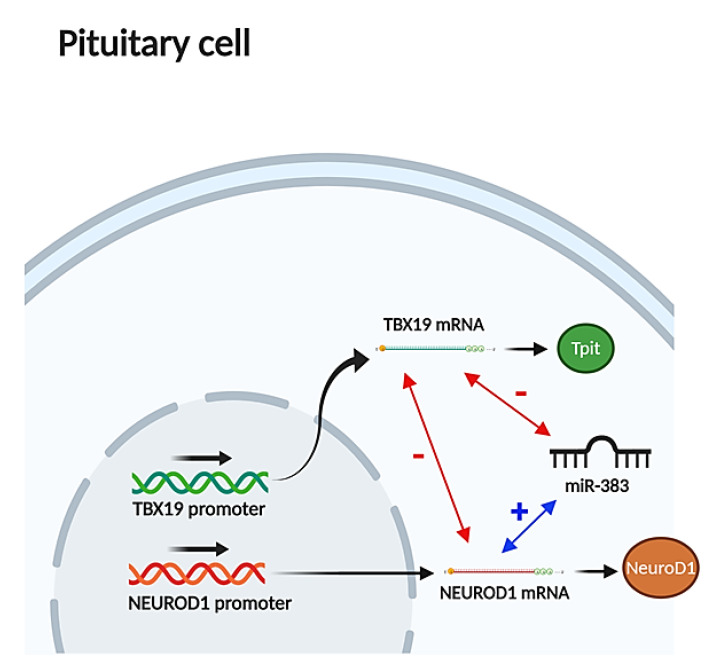
Correlations among *TBX19*, *NEUROD1* and miR-383. According to this scheme, miR-383 could negatively modulate the expression of the transcription factor of the corticotroph lineage, Tpit, through the direct inhibition of *TBX19* mRNA and by the indirect stimulation of *NEUROD1* mRNA.

Fortunately, the expression levels of *TBX19* and *NEUROD1*, two transcription factors related with the processing of *POMC*, were available in the CT samples included in this study [11]. Therefore, we could compare the relationship between miR-383 and the expression of these two key genes. We found a negative correlation between miR-383 and *TBX19* and a positive correlation with *NEUROD1* in both functioning and silent CTs: *TBX19*, silent CTs rho = −0.583, *p* = 0.007 and functioning CTs rho = −0.470, *p* = 0.049; *NEUROD1*, silent CTs rho = 0.647, *p* = 0.005; functioning CTs rho = 0.445, *p* = 0.05. We found that *NEUROD1* is a potential target for miR-383 using the three databases screened (Supplementary Table S2) according to the different computational algorithms used (Figure 3).

### 3.3. Expression Levels of miRNAs and Correlation with the Tumor Size of CTs

**Figure 4:**
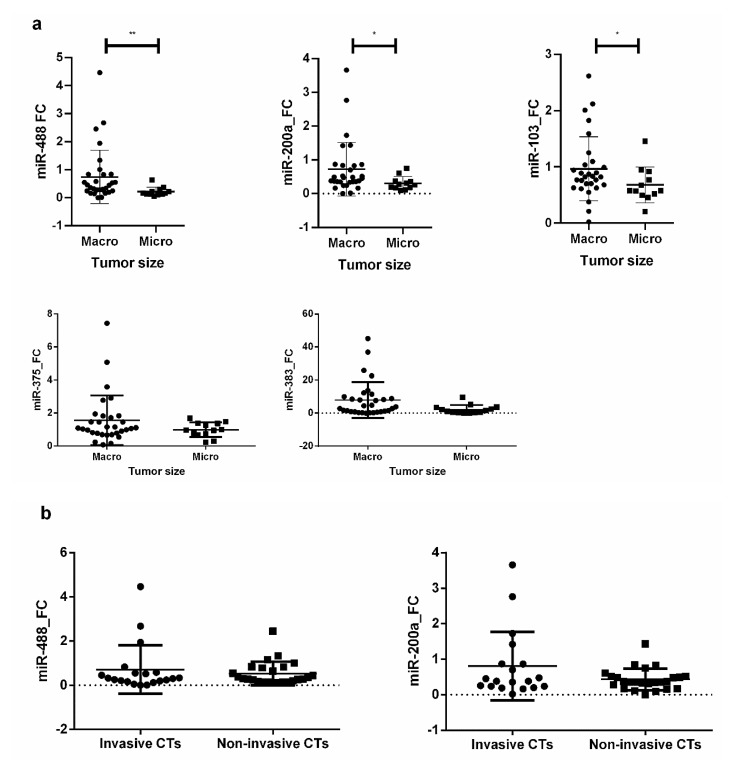
(**a**) Gene expression differences (fold change) of miR-488, miR-200a, miR-103, miR-375, and miR-383 between macro and micro corticotroph tumors; (**b**) gene expression differences of miR-488 and miR-200a between invasive and non-invasive corticotroph tumors. * *p* < 0.05; ** *p* < 0.01.

Silent CTs were significantly larger than functioning CTs (mean MTD ± standard deviation (SD): 22.44 mm ± 10.26 vs. 13.76 mm ± 9.99, *p* = 0.003). The gene expression levels of three miRNAs (miR-488, miR-200a, and miR-103) were significantly higher in macroadenomas (both functioning and silent) than in microadenomas (all functioning): miR-488 (*p* = 0.003), miR-200a (*p* = 0.020), and miR-103 (*p* = 0.049). Contrarily there were no differences in the expression levels of miR-375 and miR-383 (Figure 4a).

Indeed, there was a positive correlation between MTD and both miR-488 and miR-200a expression in the global series (rho = 0.472, *p* = 0.003 and rho = 0.420, *p* = 0.010, respectively) and in silent CTs (rho = 0.435, *p* = 0.035 and rho = 0.594, *p* = 0.005, respectively). However, there were not significant differences in the expression of both miRNAs between invasive and non-invasive corticotroph tumors (Figure 4b).

## 4. Discussion

The present study analyzes the expression of a subset of transcription factors, kinases, and miRNAs related with the melanocortinergic pathway and with proliferation, differentiation, and transcription regulation in a large series of functioning and silent CTs. Approximately 2000 miRNAs have been identified in humans, and these seem to regulate a third of the genes in the human genome [27]. Furthermore, miRNAs have been shown to play a relevant role in many biological processes. Indeed, the altered miRNA expression has been involved in the pathogenesis of numerous cancers. As shown in Supplementary Table S3, in PitNETs different miRNA expression patterns and potential targets of these miRNAs have been described, depending on the tumor subtypes [28,29,30]. In this way, our research provides new information on the potential role of miRNA on the silencing mechanisms in some corticotroph tumors that do not produce Cushing syndrome. Among the most relevant results obtained is the differential expression of miR-200a and miR-103 in silent CTs compared with functioning CTs, with a potential usefulness in the discrimination of both variants. Additionally, our results indicate that miR-383 is overexpressed in CTs, especially in silent CTs, and correlated negatively with *TBX19*, a transcription factor related with the proccesing of *POMC.*

### 4.1. miRNAs as Potential Diagnostic Biomarker of Silent CTs

As shown, miR-200a and miR-103 exhibited significantly higher expression in silent than in functioning CTs, even after eliminating the bias of tumor size, enabling the differentiation between the two variants. Given their stability in extracellular fluids, there is increasing interest in the use of miRNAs as circulating biomarkers [31], and correlations have been observed between the expression of miRNAs in serum or plasma and their expression in tumors [31,32]. However, no circulating miRNAs are being employed in the case of PitNETs. As Di Leva et al. reported in 2014 [33], since the pituitary is highly vascularized and releases hormones into the circulation, extracellular miRNAs would be useful as diagnostic biomarkers of PitNETs. Due to the distinct aggressive behavior of the PitNETs subtypes, the possibility of identifying its silent counterparts in a non-invasive way could be of great help to plan the surgical intervention. So, the tumor overexpression of miR-200a and miR-103 and the accuracy we observed in discriminating silent from functioning CTs, encourages the study of the diagnostic accuracy of its determination in peripheral blood before surgery.

### 4.2. Silencing Role of miRNAs in CTs

There is little data describing the role of miRNAs in the silencing mechanisms of PitNETs. Recently, Neou et al. [23] analyzed an important series of 134 PitNETs, 35 of whom were corticotroph tumors, finding that CT tumors were arranged in two clusters with different miRNA signatures, with clear differences between functioning tumors and their silent counterparts. The higher expression levels of miR-200a and miR-103 demonstrated in this study suggest that miRNAs could be involved in the silencing of this clinically relevant PitNET subtype. Since all microadenomas were functioning CTs and all silent CTs were macroadenomas, there is a bias regarding to their size. Therefore, we only compared functioning and silent macroadenomas. Excluding functioning microadenomas, miR-200a and miR-103 continued to be differentially expressed in both variants. Indeed, in silent but not in functioning CTs, we observed a strong negative correlation between miR-200a and miR-103 and the expression of a key functional gene in this PitNET subtypes such as *MAP3K8*. Moreover, miR-103 also correlated negatively with the expression of *MEK*. As previously described, the effect of CRH on *POMC* transcription is mediated by the *MAPK* pathway, which implicates different genes, such as *MAP3K8* and *MEK* [14,34]. For that reason, miR-200a and miR-103 could be functionally connected with the *POMC* gene by inhibiting its expression. Indeed, in our study, both miRNAs appeared down-regulated in functioning but normo-regulated in silent CTs. 

In addition, our results revealed that other miRNAs, such as miR-375, miR-488 and miR-383, could also be directly involved in the silencing of CTs. Indeed, we found a higher expression of miR-375 in silent CTs than in functioning ones, and it has been described that *MAP3K8* is a direct target gene of miR-375 in AtT-20 mouse pituitary tumor cells [14]. Moreover, we observed that miR-488 was down-regulated and presented a negative correlation with *NURR1*, *PITX1*, and *STAT3* (all *POMC* expression enhancers) in functioning CTs. Therefore, these results might suggest that miR-488 could exert a permissive effect on the expression of *POMC* in functioning CTs, probably through the *STAT3* pathway [15,35]. In the same way, miR-383 was especially up-regulated in silent CTs and, as it showed a negative correlation with *STAT3*, this miRNA might also participate in the silencing mechanisms of CTs [16]. Indeed, we found that miR-383 correlated negatively with *TBX19*, a transcription factor necessary for the differentiation and maintenance of adult corticotroph cells [36,37] (Figure 3). Finally, as somatostatin receptor genes (*SSTR2*, *3* and *5*) are putative targets for miR-383, and *STAT3* has been proposed as a promising target candidate for pituitary tumor immunotherapy [38], our results might also suggest that miR-383 could be a therapeutic target in corticotroph PitNETs.

### 4.3. miRNAs: Tumor Size and Proliferation

Several miRNAs have been associated with PitNET size and growth [29]. In 2005, Bottoni et al. observed a negative correlation between tumor diameter and expression of miR-15a and miR-16-1 in somatotroph and lactotroph tumors [17]. Later, the same group described six miRNAs able to differentiate microadenomas from macroadenomas in non-secreting tumors [18]. In the same way, Butz et al. suggested that miR-629 and miR-214 may contribute to tumor growth in non-secreting pituitary tumors through an impaired regulation of apoptosis [39]. Finally, Renjie at al. demonstrated a tumor suppressor role of miR-15a, miR-16, and miR-132 in pituitary tumor cells (GH3 and MMQ cells) and in most PitNETs [40]. However, to the best of our knowledge, none of these studies has focused on the analysis of miRNAs and key regulatory genes in silent and functioning CTs. Specifically, our study revealed that miR-488, miR-200a, and miR-103 showed higher expression levels in macroadenomas than in microadenomas. As all microadenomas were functioning CTs and all silent CTs were macroadenomas, we focused on silent CTs. Thus, we found that miR-488 and miR-200a expression levels were positively correlated with tumor size, suggesting that these miRNAs could participate in the growth of these tumors. Our results are concordant with other studies reporting that miR-488 and miR200 play a role as tumor suppressor agents in several types of cancers [41,42,43,44,45,46,47]. In addition, we observed a strong negative correlation between the expression of *MAP3K8* and miR-488, miR-200a and miR-103, and also between the expression of *MEK* and miR-488 and miR-103 in silent CTs. As *MAP3K8* acts as an oncogene in numerous cancer types [48,49,50] and the *MEK* gene is also involved in a wide variety of cellular processes such as proliferation [51,52], the down-regulation in the expression of these miRNAs could facilitate the oncogenic effects of *MAP3K8/MEK* in the growth of CT PitNETs. These observations unveiled new conceptual and functional avenues in CTs, with potential therapeutic implications, which warrant further investigation.

## 5. Conclusions

In conclusion, the evidence from this study suggests that miRNAs could participate in the silencing and growth of CTs. Specifically, we demonstrated that miR-200a, miR-103, miR-375, miR-488 and miR-383 could participate in the silencing mechanisms of these clinically relevant tumors, which are not associated with Cushing syndrome. In addition, miR-488, miR-200a and miR-103 could also be involved in the growth of CTs. Altogether, the translational research implications of these findings indicate that these miRNAs might be involved in the pathophysiology of CTs, suggesting that pharmacological treatments specifically targeting these miRNAs could become a promising therapeutic option for patients in the future, and provide a relevant clinical conclusion, which should soon be tested in humans.

## Figures and Tables

**Table 1:** Demographic and clinical characteristics of the studied cohort.

		Gender		Age	Tumor Size		Invasiveness		MTD
Subtype	n (%)	Women	Men	Years	Macro	Micro	Yes	No	mm
		n (%)		Mean ± SD	n (%)		n (%)		Mean ± SD
Functioning CTs	24 (51.1)	20 (83.3)	4 (16.7)	43.71 ± 11.74	11 (45.8)	13 (54.2)	7 (29.2)	17 (70.8)	13.87 ± 10.19
Silent CTs	23 (48.9)	16 (69.6)	7 (30.4)	43.30 ± 15.04	23 (100)	0 (0)	15 (65.2)	8 (34.8)	21.61 ± 9.21

MTD, maximum tumor diameter; SD, standard deviation.

## Supplementary Materials

The following are available online at https://www.mdpi.com/2077-0383/9/6/1838/s1, Figure S1: Receiver operating characteristics (ROC) curves of miR-200a and miR-103. Table S1: Corticotroph tumors; Table S2: TargetScan, miRBase and PicTar results; Table S3: Main Published Studies on miRNAs expression in funcioning and non-functioning CTs.
