# Supplementary material for: Differential Expression of MicroRNAs in Silent and Functioning Corticotroph Tumors
Source: J Clin Med. 2020 Jun 12;9(6):1838. doi: 10.3390/jcm9061838 (PMC7355784; doi:10.3390/jcm9061838)
Supplement: Supplementary file 1 [file jcm-09-01838-s001.zip › jcm-807529suppl/jcm-807529Table S3.docx]

**Supplementary Table 3.** Main Published Studies on miRNAs expression in funcioning and silent CTs.

| **Author (year)** | **Samples** | **Genes and miRNAs** | **Techniques** | **Conclusions** |
| --- | --- | --- | --- | --- |
| Bottoni (2007) | 6 STs, 5 LTs, 4 CTs, 17 silent PitNETs | 245 miRNAs | miRNA array  qRT-PCR (TaqMan, relative expression) | 7 upregulated and 23 downregulated miRNAs in different PitNET subtypes  29 miRNAs capable of predicting the histotype  6 miRNAs differentially expressed in macro and micro silent PitNETs |
| Amaral (2009) | 14 sCTs | let7a, miR-15a, miR-16, miR-21, miR-141, miR-143, miR-145, miR-150 | qRT-PCR (TaqMan, relative expression) | 8 miRNAs under-expressed in secreting CTs |
| Liang (2013) | 10 silent PitNETs, 10 GTs, 2 normal pituitary gland | 435 human miRNAs | miRNA array  qRT-PCR (SYBR green, relative expression) | 16 upregulated and 13 downregulated miRNAs in GTs |
| Palumbo (2013) | 7 STs, 5 normal pituitary gland, AtT20 cells | 365 miRNAs | miRNA TaqMan array  qRT-PCR (SYBR green, relative expression) | 5 upregulated (miR-26a, miR-26b, miR-212, miR-107, miR-103) and 12 downregulated (miR-125b, miR-141, miR-144, miR-164, miR-145, miR-143, miR-15b, miR-16, let7b, let7a3, miR-128) in STs  miR-26b and miR-128 regulate *PTEN-AKT* pathway in AtT20 cells  Negative correlation between miR-26b/miR-128 and PTEN and between miR-128 and *BMI1* in STs |
| Gentilin (2013) | 5 CTs, AtT20 cells | miR-26a, miR-150, miR-212, miR-191, miR-181b, miR-98, miR-189, miR-24, miR-192, miR-124a, miR-153 | qRT-PCR (TaqMan, relative expression)  Functional studies | miR-124a, miR-24, miR-191 and miR-121 downregulated and miR-181b and miR-26a upregulated in AtT20 cells  *PRKCD* and *SENP5* putative target genes of miR-26a |
| Garbicz (2017) | 25 PitNETs (sCTs and silent CTs), 4 Crooke’s cell | miR-106b-5p, miR-93-5p, miR-93-3p, miR-25-3p, *MCM7* | qRT-PCR (TaqMan, relative expression)  IHC | miR-106~25 upregulated in invasive ACTH-producing PitNETs |
| Neou (2020) | 134 PitNETs (35 CTs, 29 GTs, 23 STs, 16 LTs, 8 mixed GH-PRL, 8 NC, 6 TT, 9 PH-PIT1) | miRNome | miRNA sequencing | CT tumors were arranged in two clusters with different miRNA signatures, with clear differences between functioning and silent variants. The most differentially expressed miRNA between the miRNome groups were: miR-410-3p, miR-148ª, miR-577, miR-934, miR-132/miR-212, miR574, MEG3-cluster, miR-656-3p, miR-208b-3p, miR-582, miR-1468-5p, miR-532/let-7f-2, miR-28, miR-137 and miR-504-5p |
| Present study (2020) | 47 PitNETs (24 sCTs and 23 silent CTs) | miR-488, miR-200a, miR-103, miR-383, miR-375  *PKA, MAP3K8, MEK, MAPK3, NGFIB, NURR1, PITX1, STAT3* | qRT-PCR (TaqMan, relative expression) | miR-200a and miR-103 present potential usefulness in the discrimination of secreting and silent CTs. miR-383 overexpressed in CTs especially in silent CTs. Negative correlation between *TBX19* and miR-383 in silent CTs |
