# Supplementary figures and images for: Differential Expression of MicroRNAs in Silent and Functioning Corticotroph Tumors
Source: J Clin Med. 2020 Jun 12;9(6):1838. doi: 10.3390/jcm9061838 (PMC7355784; doi:10.3390/jcm9061838)

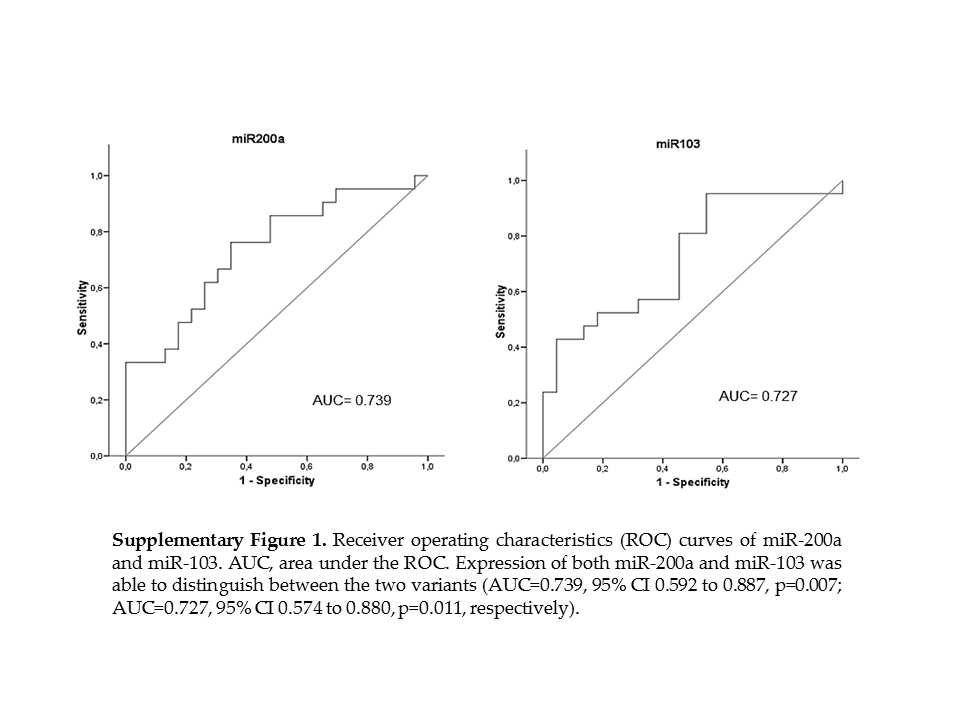

Supplement: Supplementary file 1 [file jcm-09-01838-s001.zip › jcm-807529suppl/Supplementary Figure 1.tif]
